# Use of qPCR to monitor 2,4-dinitroanisole degrading bacteria in water and soil slurry cultures

**DOI:** 10.1093/jimb/kuae047

**Published:** 2024-11-23

**Authors:** Lisa A Waidner, Carrie E Daniel, Sarah E Kovar, Jim C Spain

**Affiliations:** Center for Environmental Diagnostics and Bioremediation, University of West Florida, 11000 University Pkwy, Building 58, Pensacola, USA; Center for Environmental Diagnostics and Bioremediation, University of West Florida, 11000 University Pkwy, Building 58, Pensacola, USA; Center for Environmental Diagnostics and Bioremediation, University of West Florida, 11000 University Pkwy, Building 58, Pensacola, USA; Center for Environmental Diagnostics and Bioremediation, University of West Florida, 11000 University Pkwy, Building 58, Pensacola, USA

**Keywords:** Insensitive munitions compounds (IMCs), 2,4-dinitroanisole (DNAN), qPCR, Molecular tools, Biodegradation

## Abstract

Prediction and process monitoring during natural attenuation, bioremediation, and biotreatment require effective strategies for detection and enumeration of the responsible bacteria. The use of 2,4-dinitroanisole (DNAN) as a component of insensitive munitions leads to environmental contamination of firing ranges and manufacturing waste streams. *Nocardioides* sp. strain JS1661 degrades DNAN under aerobic conditions *via* a pathway involving an unusual DNAN demethylase. We used the deeply branched sequences of DNAN degradation functional genes as a target for development of a molecular method for detection of the bacteria. A qPCR assay was designed for the junction between *dnhA* and *dnhB*, the adjacent genes encoding DNAN demethylase. The assay allowed reproducible enumeration of JS1661 during growth in liquid media and soil slurries. Results were consistent with biodegradation of DNAN, accumulation of products, and classical biomass estimates, including most probable number and OD_600_. The results provide a sensitive and specific molecular method for prediction of degradation potential and process evaluation during degradation of DNAN.

**One-Sentence Summary:**

A unique target sequence in functional genes enables the design of a simple and specific qPCR assay for enumeration of aerobic 2,4-dinitroanisole-degrading bacteria in soil and water.

## Graphical Abstract

**Graphical Abstract:**
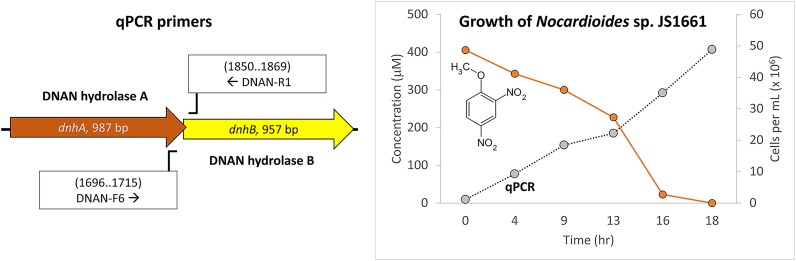

## Introduction

The growing use of insensitive munitions compounds (IMCs), such as 2,4-dinitroanisole (DNAN) and 3-nitro-1,2,4-triazol-5-one (NTO), leads to environmental contamination at firing ranges and problems with disposal of waste streams at manufacturing facilities. IMCs were developed >40 years ago (Powell, 2016), but have been widely used only in the past decade. The concentrations of DNAN in manufacturers’ waste streams can range from ∼110 to 150 mg L^−1^ (Felt et al., 2013; Shen et al., 2013; Schroer et al., 2017; Hadnagy et al., 2018; Fawcett-Hirst et al., 2021). Environmental contamination of water and soils with these munitions compounds is problematic because of potential toxicity and mutagenicity to humans and other organisms (Liang et al., 2013; Madeira et al., 2018; Purohit and Basu, 2000; Menezes et al., 2022a). A variety of biotic and abiotic DNAN transformations have been reported (Weidhaas et al., 2018; Niedźwiecka et al., 2020; Menezes et al., 2021; Wang et al., 2024), but ideal bioremediation or natural attenuation strategies should include DNAN biodegradation (mineralization) under environmentally-relevant conditions.

Biodegradation of DNAN under aerobic conditions has been established in *Nocardioides* sp. strain JS1661 which was isolated from activated sludge at Holston Army Ammunition Plant (Fida et al., 2014). DNAN is biodegraded by strain JS1661 in minimal media, soil, and bioreactors (Karthikeyan & Spain, 2016; Menezes et al. 2022b), and JS1661 grows with DNAN as the sole carbon source as previously described (Fida et al., 2014). Initial attack on DNAN by a demethylase enzyme produces 2,4-dinitrophenol which is then degraded *via* a series of reactions resulting in release of nitrite and accumulation of biomass (Fida et al., 2014). In mixed microbial communities such as those found in soil and bioreactors, nitrite is subsequently oxidized to nitrate by nitrifying bacteria (Karthikeyan & Spain, 2016; Menezes et al., 2022b).

Prediction and process monitoring during natural attenuation, bioremediation, and biotreatment require effective strategies for detecting the biodegrading microbes. The population density and growth must be determined to be consistent with the biodegradation rates (ASTM, 2022). Enumerating specific strains in microbial communities using 16S rRNA genes is limited because of variable number of 16S rRNA operons per cell (Zemb et al., 2020), highly similar 16S rRNA sequences, even within hypervariable regions (Chakravorty et al., 2007), the need for a third oligonucleotide, e.g., TaqMan probe (Chakravorty et al., 2007; Ritalahti et al., 2006) or a nested 16S PCR assay design which may limit the quantification range (Ritalahti & Löffler, 2004; Löffler et al., 2000; Marušincová et al., 2013).

Possible molecular methods include metagenomics, amplicon sequencing for the 16S rRNA gene, or other methods targeting known functional genes for the biodegradation processes. Using metagenomics analysis of municipal anaerobic digester sludge, growth of dominant community members with known functional capability was observed simultaneously with the observed reduction of the NTO contaminant (Madeira et al., 2021). Similarly, myriad other studies evaluate full community structures of biodegradative communities, sometimes supplementing findings with genome sequencing of isolates or quantitative PCR (qPCR) for specific functional genes (Kharey et al., 2020; Chen et al., 2021; Richards et al., 2022; Dang et al., 2023). The drawback of 16S rRNA gene sequencing is that the catabolic pathways are not uniformly present in all members of the species, as recently described for metaldehyde biodegradation potential (Castro-Gutierrez et al., 2022). Currently, among the variety of molecular methods, qPCR is still considered the “gold standard” technique for tracking functional genes in biodegrading microorganisms in samples containing an environmental contaminant. Those using multiplexing or TaqMan probe designs for functional genes include biomarkers for 1,4-dioxane metabolism, *dxmB* (Miao et al., 2020), anaerobic toluene degradation, *bssA* (Pilloni et al., 2019), as well as multi-gene assay designs for RDX biodegradation (Wilson & Cupples, 2016; Collier et al., 2019). The use of qPCR provides an advantage in sensitivity, specificity, wide range of detection, and lower cost than with other molecular biological methods (ASTM, 2022).

Specific qPCR assays for functional genes involved in a range of biodegradation pathways are commercially available (ASTM, 2022). Unfortunately, some require multiplexed qPCR for multiple targets (Inoue et al., 2020; Yang et al., 2023), degenerate bases in qPCR primers to target diverse functional gene sequences (Jin & Mattes, 2010), or the use of TaqMan probes in multiplexed qPCR assays, i.e., combining several single-target primer pairs (Collier et al., 2019; Dang & Cupples, 2021). Probe-based or multiplex qPCR assays are described for a wide variety of biodegradation functional genes, including for soil pesticide metaldehyde degradation (Castro-Gutierrez et al., 2022), for 1,4-dioxane-degrading bacteria (Inoue et al., 2020) and for soil 4-chlorobenzoate biodegradation potential (Rodrigues et al., 2002). Such multiplex qPCR combination or probe-based assay types are relatively expensive as compared to a SYBR Green-based assay as described in this report. The most economical assay for detecting the specific functional genes would involve a single set of non-degenerate qPCR primers with high specificity. Although complex strategies are required for distinguishing among closely-related strains, the approach can be more straightforward when there are unique or deeply-branching functional genes.

The goal of this work was to develop a molecular method for enumeration of bacteria with the capacity for aerobic DNAN biodegradation. The candidate biomarker employed here, the *dnhA–dnhB* gene pair, is specific to the only known DNAN degrader, *Nocardioides* sp. strain JS1661 (Fida et al., 2014).

## Materials and Methods

### Growth in Liquid Media

Liquid cultures of JS1661 were grown in ½ strength minimal salts (½ MSB) media (Cohen-Bazire et al., 1957), pH 6.5, and supplemented with DNAN to final concentrations of approximately 400 µM as described previously (Fida et al., 2014; Karthikeyan & Spain, 2016). Cultures were grown at 30°C with shaking at 100 rpm in duplicate flasks. At appropriate intervals, samples were removed for DNA extraction, most probable number (MPN) analyses, nitrite, optical density (OD_600_), and High Performance Liquid Chromatography (HPLC) analyses.

### Growth in Soil Slurry

The soil slurry cultures of JS1661 contained low organic, dried sandy loam soil that was sieved (30–40 mesh) as described previously (Karthikeyan & Spain, 2016). Soil (10% w/v) was suspended in ½-strength MSB containing DNAN (∼350 µM) then inoculated, incubated, and analyzed as described above. Each soil slurry was 150 mL, composed of 15 grams soil, 10 mL of culture inoculum, and 125 mL of media. Suspensions were stirred on a magnetic stir plate during sampling with wide bore pipette tips.

### MPN

From duplicate flasks, samples for MPN estimations were sonicated for 5 s and then subjected to serial 10-fold dilutions in ½ MSB pH 6.5 supplemented with DNAN (400 µM). Sixteen 200 µL samples from each dilution were added to 96 well round bottom microplates which were then sealed and incubated at 30°C. Each microplate thus constituted 2 sets of 8-tube MPN’s. After 10 days, growth was scored by accumulation of visible pellets in the bottom of the wells. The EPA MPN calculator using the Cornish & Fisher Limits, with a confidence interval of 95% (EPA, 2024) was used for estimation of viable cells per mL in the liquid culture or soil slurry experiments.

### DNA Extraction and Analysis

For DNA extraction from liquid cultures and soil slurries, biomass was harvested by filtration and centrifugation, respectively. Samples from liquid cultures (10 mL) were filtered (Durapore^®^ GV 0.22 µm, 25 mm diameter, EMD Millipore, Burlington, MA, USA). Filters with biomass were each transferred to bead-beating tubes with 0.1 g each of 0.5 mm and 0.1 mm Zirconia/silica beads plus 500 µL of lysis buffer (20 mM Tris-HCl, pH 8, 2 mM EDTA, 200 mM NaCl, and 0.2% Triton X-100). Bacterial biomass from slurries was harvested by centrifugation (10 min, 20 800 *g*, 5°C), supernatants were discarded, and pellets were mixed with beads and lysis buffer. Harvested biomass in both procedures was stored at −20°C until DNA was extracted.

A modified method of the Qiagen Gram-positive DNA extraction protocol (QIAamp^®^ DNA Mini, #51 306, Qiagen, Hilden, Germany) was used for all samples and the for the qPCR positive control culture. Modifications included enzymatic digestion with lysozyme (in addition to Proteinase K), bead beating, and RNA removal with RNAse A. Lysozyme was added to thawed samples (final concentration 2.8 mg mL^−1^), and incubated at 37°C for 30 min. Physical cell disruption was done for 2 × 1.0 min at a setting of 4.2 (Bead Ruptor Elite model, OMNI International, Kennesaw, GA, USA). RNA was removed with RNAse A (1.0 mg mL^−1^) at 24°C for 2 min, and then the rest of the manufacturer’s protocol steps were followed. Except for standard DNA isolated from pure *Nocardioides* sp. strain JS1661, DNA preparations were used undiluted in qPCR.

### Quantitative PCR

Initial qPCR primer testing was with conditions as described below, followed by end-point analysis of amplicon purity and size *via* gel electrophoresis (2% agarose in 1X TAE, Supplemental Fig. S2). Additional amplicon validation was done on a preparation of end-point product subjected to electrophoresis and excision from the gel, then purification (QIAquick Gel Extraction, Qiagen, Hilden, Germany). The purified PCR product was subjected to Sanger sequencing in both directions (GeneWiz, Azenta Life Sciences, South Plainfield, NJ, USA).

Primer pair DNAN-F6 and DNAN-R1 (Table 1) was used in qPCR run in an Applied Biosystems™ QuantStudio™ 3 instrument (Thermo Fisher Scientific, Waltham, MA, USA). Initial 95°C incubation was for 1 min, followed by 40 cycles of 95°C (15 s), 60°C (60 s), and 72°C (20 s), with detection normalized to ROX during extension, and completed with a melt curve step. Melt curves were evaluated in each run, but amplicon size was also validated by electrophoresis (see above). Reactions were composed of 0.08 µM each forward and reverse primers, 1X Applied Biosystems™ PowerTrack™ SYBR Green master mix (Thermo Fisher Scientific, Waltham, Mass.), 2 µL of purified DNA sample, and water to achieve a 20 µL final volume.

**Table 1.** Primers for qPCR Enumeration of *dnhA–dnhB* Genes

Primer name	NN Tm (°C)^A^	Position		Sequence (5′ to 3′)
		5′ base^B^	3′ base^B^	
DNAN-F6	55.3	1 696	1 715	TACGAGCCGTACTCCAACGA
DNAN-R1	55.8	1 869	1 850	TTCGATACCCGTCGATTGCG

A Nearest-neighbor melting temperature.

B Position in genomic DNA segment containing the *dnhA-B* genes, (accession# KM213001.1), as previously described (Fida et al., 2014).

DNA for standard curves was isolated from *Nocardioides* sp. strain JS1661 pure cultures and used for calculations of absolute gene quantities in experimental samples (Supplemental Fig. S2). Standard DNA was assessed by UV spectrophotometry (NanoDrop^TM^ ND-1000 V3.8.1, ThermoFisher Scientific, Waltham, MA, USA), and 10-fold serial dilutions in 10 mM Tris-1 mM EDTA (T_10_E_1_) were used in qPCR. All qPCR measurements were done in triplicate.

### HPLC

HPLC was used to determine DNAN and 2,4-DNP concentrations. Liquid or slurry samples were mixed with equal volumes of acetonitrile, vortexed, clarified by centrifugation, and analyzed by HPLC. Chromatography was done with an Agilent 1260 HPLC system using a Chromolith high resolution RP18 150–4.6 mm column. The mobile phase was 50% acetonitrile with 0.1% trifluoroacetic acid and 50% water with 0.1% trifluoroacetic acid. Absorbance of DNAN and 2,4-DNP were monitored at 300 nm with an Agilent Diode Array Detector (Model G4212B).

## Chemicals

DNAN (CAS # 119‐27‐7, purity = 98%), was from Alfa Aesar (Ward Hill, MA, USA), 2,4-DNP (CAS # 51‐28‐5, purity ≥ 98%) was from Sigma–Aldrich (Millipore-Sigma, St. Louis, MO, USA), and all other chemicals were reagent grade or better.

### Nitrite and OD_600_ Assays

Nitrite was measured as described previously (Daniels et al., 2007), using the Griess method and measurement of final absorbance at 543 nm. All absorbance measurements were done in 96 well plates in a microplate reader (Synergy HT, Biotek, Santa Clara, CA, USA).

### DNA Sequence Analyses

Genome sequences were annotated and compared in RAST (Aziz et al., 2008). Additional manual curation to evaluate percent identity of amino acid sequences among homologs was performed with BLASTp or BLASTn analyses (Altschul et al., 1997). For primer design, nearest-neighbor melting temperatures (NN Tm) were evaluated as previously described (Kibbe, 2007). Sanger sequencing reads were edited in SnapGene Viewer, and the consensus sequence was formed in MEGA-X (Kumar et al., 2018). The resulting 174-nucleotide sequence was aligned with the previously-published *dnhA-B* gene sequences to validate the expected amplicon.

### JS1661 Draft Genome

The draft genome of *Nocardiodes* sp. strain JS1661 was obtained by sequencing as previously described (Fida et al., 2014). Briefly, Illumina sequencing (HiSeq 2000) was followed by *de novo* read assembly (Tritt et al., 2012) and annotation with RAST (Aziz et al., 2008). A single copy of the *dnhA-B* genes is in the ∼5.8 Mbp draft genome. The 4 993 bp annotated contig containing the *dnhA-B* genes as shown in Supplemental Fig. S1 is publicly available (accession #KM213001.1), as previously described (Fida et al., 2014).

## Results and Discussion

### *dnhA-B* qPCR Assay design, Sensitivity, and Specificity

In *Nocardiodes* sp. strain JS1661, the DNAN demethylase is encoded by two genes whose open reading frames overlap by 3 nucleotides. Base positions # 984–987 (GTGA) of the 987-bp *dnhA* gene overlap with the first four bases of the 957-bp open reading frame for *dnhB*, starting with the alternative GTG codon (Supplemental Fig. S1, panel a). To date (August 2024), there are no functionally characterized protein sequences that are closely related to the DNHA and DNHB proteins (Supplemental Table S1). Most homologs have amino acid identity <60% and are open reading frames with unknown function from metagenome-assembly genome (MAG) segments. Two characterized proteins include the Zn-bound alkylsulfatases from *Escherichia coli* (Liang et al. 2014) and *Pseudomonas* sp. (Knaus et al., 2012), but percent identity is low, and only the central regions of each align with portions of the DNHA or DNHB sequences.

The one cultured strain that contains homologs to *dnhA-B* in tandem is *Nocardia testacea* NBRC 100365 (Supplemental Table S1 and Supplemental Fig. S1), but the *Nocardia* proteins have not been functionally characterized. The B homolog is annotated as a hypothetical protein, and the A homolog is a putative member of the metallo-beta-lactamase (MBL) fold metallo-hydrolase superfamily. Most of the JS1661 DNHA homologs are annotated as MBL fold hydrolases, containing the established conserved domain database sequences (Wang et al., 2023; NCBI CDD, MBL-fold, 2024). The DNAN hydrolase subunits DNHA and DNHB seem to constitute a deeply-branching subclass within the superfamily (Supplemental Fig. S3).

The nucleotide sequence at the junction of JS1661 *dnhA-B* is unique. The region targeted by our qPCR assay has little to no nucleotide identity with the DNA sequences encoding the A and B homologs in *N. testacea* or any of the more distantly related homologs. In the regions where we designed qPCR primers, the homolog sequences’ identities are so low (<50% of 20 bases) that primer annealing would not occur under standard qPCR conditions. The 174-bp amplicon is a product including the end of *dnhA* and the start of *dnhB* (Table 1 and Supplemental Fig. S1, panel b). When primers were initially tested in PCR with DNA from a pure culture of strain JS1661, the resulting amplicon was of the anticipated length (Supplemental Fig. S2, panel a). Sequencing of the amplicon verified that it matched with 100% nucleotide identity to the *dnhA-B* junction (positions #1696–1869 of accession #KM213001.1).

The assay gave reproducible detection down to ∼100 gene copies (Supplemental Fig. S2, panel b). Cycle threshold (*C*_T_) values ranged from ∼18 to 36, well below the 40 cycles used to assess amplicon size and purity described above. An attempt to detect ∼10.6 copies per reaction was also done (not shown), but a measurable *C*_T_ value (39.24) was observed in only one of three wells. In 100% of the standard curve qPCR experiments performed, there were no “undetectable” wells in qPCR assays containing 106 gene copies. The calculated lower detection limit of the assay from replicate standard curves, including 24 wells containing the lowest concentration of the DNA standard, was 38.19 copies per reaction, with a confidence interval of 95%. In all experimental samples and standard curves, the upper limit assayed was ∼1.6 × 10^6^ copies (Supplemental Fig. S2, panel b). We also evaluated assay precision for all experimental samples and standard DNA reactions, and replicate error was low in all cases. In all experimental DNA samples from liquid and soil slurry cultures, the assay coefficient of variation (CV) ranged from 0.1% to 11%, with an average experimental CV of 6%. In triplicate wells for each of the standard DNA concentrations, the CV was even lower, from 0.1% to 1.5%. Therefore, qPCR abundances for duplicate flasks are reported as a single average curve from both flasks, without including the well-to-well assay error. An inhibition assessment was tested on DNA extracted from all time points of a soil slurry culture (Supplemental Fig. S5); the Ct values associated with undiluted DNA resulted in calculated gene copies of approximately ∼eight-fold higher values than those associated with qPCR on the same DNA that was diluted 10-fold.

The qPCR enumeration method was validated with DNA from both liquid and soil slurry cultures supplemented with DNAN and inoculated with strain JS1661. Consistent with previous results (Fida et al., 2014; Karthikeyan & Spain, 2016), DNAN disappearance, accumulation of nitrite, and exponential growth of strain JS1661 in liquid cultures and soil slurries was rapid and reproducible. The DNAN degradation pathway (Fida et al., 2014) indicating the role of the *dnhA-B* gene products is provided in Supplemental Fig. S4.

### Growth in Liquid Media

**Fig. 1.**
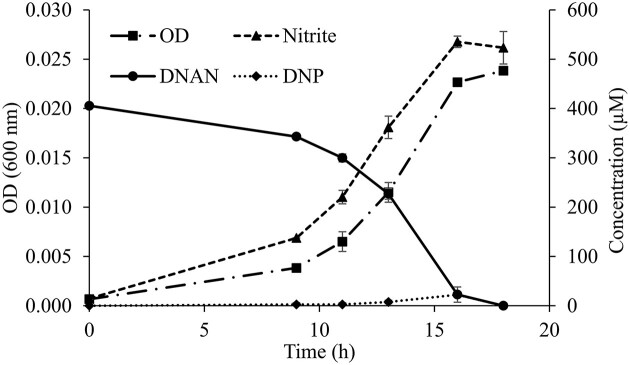
Growth of strain JS1661 and disappearance of DNAN in liquid culture. Data are mean and range of duplicate culture flasks.

**Fig. 2.**
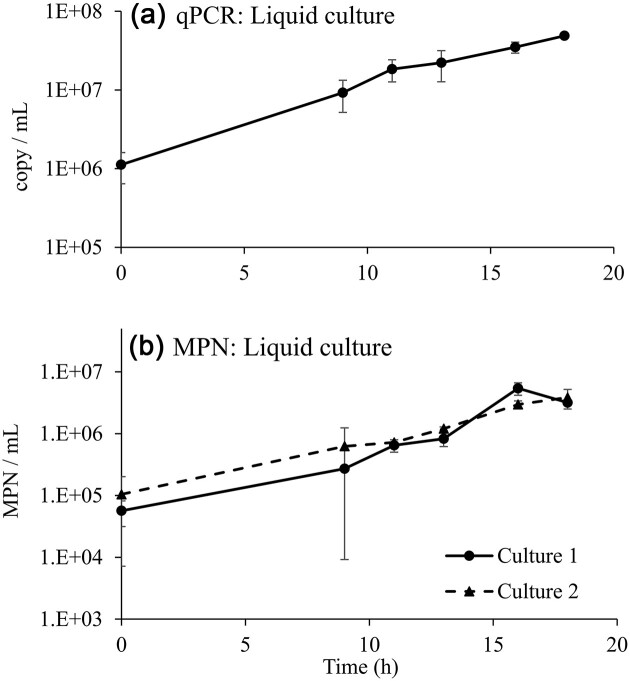
Liquid culture growth of strain JS1661 assessed by qPCR and MPN. Samples were from the cultures described in Fig. 1. The qPCR JS1661 abundance data (panel a) are means and ranges of two cultures from the liquid growth experiment. The MPN data (panel b) are plotted separately for duplicate cultures #1 and 2, where error bars represent the ranges for duplicates from each flask. The full dataset of MPN and confidence intervals for MPN assay dilutions are provided in Supplemental Table S2. Note, log scale on *Y*-axes in both panels.

When bacteria grew on DNAN in minimal media, OD_600_, MPN, and qPCR tracked growth well and were consistent with disappearance of DNAN and the transient intermediate, 2,4-DNP, along with accumulation of nitrite and biomass (Figs. 1 and 2). The qPCR assay indicated that strain JS1661 abundance increased ∼50-fold, from ∼1 × 10^6^ cells mL^−1^ to ∼5 × 10^7^ cells mL^−1^ in 18 hr (Fig. 2). It should be noted that the absorbance growth curve (Fig. 1) is an arithmetic plot, whereas the MPN and qPCR data are semilog plots. The population increase during the time course was consistent as measured by qPCR, absorbance, and MPN, but the cell abundance estimates from MPN were low compared to values obtained from qPCR. This is not unexpected, since there is a tendency of *Nocardioides* to grow in clumps or branching chains (Yoon & Park, 2006; Ma et al., 2023). The qPCR assay has advantages over traditional methods for estimating population growth. For example, abundances based on simple absorbance do not track well with other methods (e.g., CFU from dilution plating and flow cytometry), and abundance estimates can be off by as much as 10-fold depending on the cell concentration (Beal et al., 2020; Mira et al., 2022). In addition, MPN assays require the ability to grow bacteria from fresh samples, whereas qPCR can be applied with frozen samples. Finally, MPN is more tedious and is not easily integrated in workflows of commercial labs or in the field, which is why qPCR use is more widespread.

### Growth in Soil Slurries

**Fig. 3.**
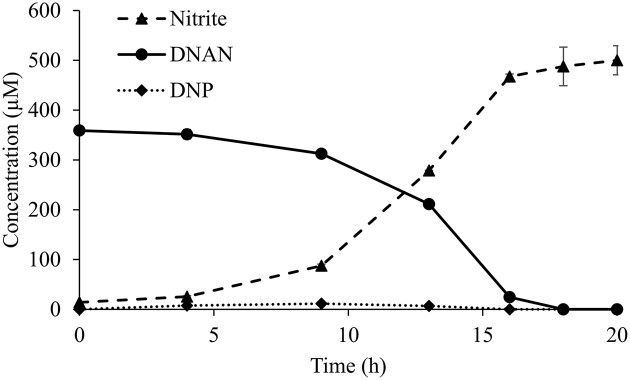
Soil slurry enrichments with strain JS1661, with disappearance of DNAN and accumulation of nitrite. Data are mean and range of duplicate culture flasks.

**Fig. 4.**
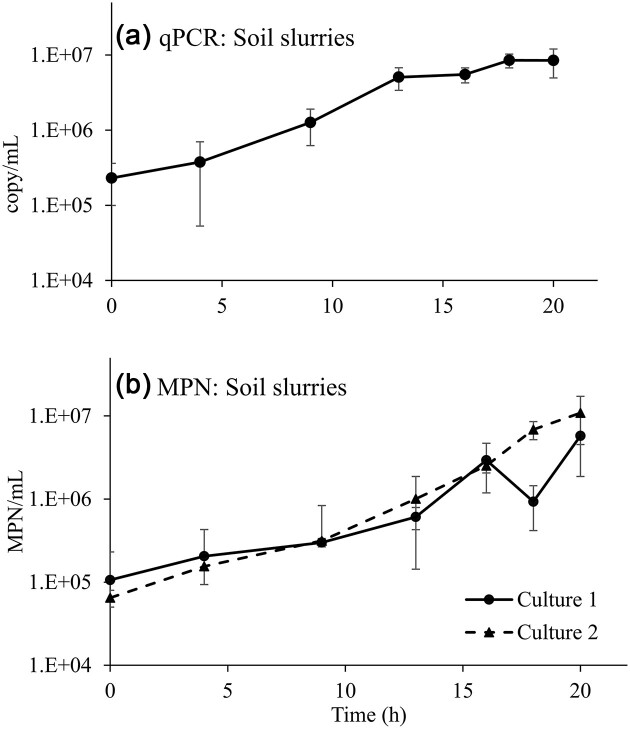
Soil slurry growth of strain JS1661 assessed by qPCR and MPN. Samples were from the cultures described in Fig. 3. The qPCR JS1661 abundance data (panel a) are means and ranges of two cultures from the liquid growth experiment. The MPN data (panel b) are plotted separately for duplicate cultures #1 and 2, where error bars represent the ranges for duplicates from each flask. The full dataset of MPN and confidence intervals for MPN assay dilutions are provided in Supplemental Table S3. Note, log scale on *Y*-axes in both panels.

A similar set of biodegradation-dependent growth experiments evaluated the ability of the qPCR assay to quantify the JS1661 population during growth on DNAN in soil slurries (Figs. 3 and 4). Because biomass estimates based on OD_600_ are not possible in soil slurries, the qPCR method was particularly important for population estimates. Consistent with the liquid cultures above, the qPCR-based abundance estimates mirrored those of the MPN data (Fig. 3). Strain JS1661 abundance increased∼40-fold in 20 hr of growth in soil slurries, based on the qPCR data (Fig. 4a). Again, as in liquid culture experiments, MPN abundances in soil slurries (Fig. 4b) were lower than qPCR estimates, likely due to attachment of the cells to soil particles (Roser et al., 1987) or *Nocardioides* growth in clumps (Yoon & Park, 2006; Ma et al., 2023). Attachment and clumping can also explain the lower precision of MPN assays reflected in the broad confidence intervals (Supplemental Tables S3–S4).

The data presented here, as well as in other reports (Liang et al., 2017; Kharey et al., 2020; Miao et al., 2020; Richards et al., 2022), add to the growing list of sensitive and specific qPCR assays for accurate measurement of bacterial growth during biodegradation of organic pollutants. However, there are currently no assays for bacteria that degrade insensitive munitions components. Because the genome size and number of copies of the functional gene target in the JS1661 genome are known, this qPCR method allowed for direct enumeration of the population. This is a particularly important consideration when evaluating abundance estimates with multiple methods, as was done here. Ideal qPCR assays should detect gene copies in a wide range of concentrations, which is not always possible, as recently reported for sulfate reduction pathway genes in sludge (Zambrano-Romero et al., 2023). In this report, the assay was tested on a wide range of copies, which is important for evaluating complex samples such as sludge, soil, and groundwater. Whereas the liquid culture tests reported here were done with pure cultures, the proof-of-concept soil slurry experiments contained naturally-occurring bacterial communities. If new DNAN degraders are isolated, their *dnhA-B* sequences can be examined, and if necessary, the qPCR primer sequences could be adjusted accordingly. Future studies should evaluate the method in a wide range of field conditions.

## Conclusions

Many pathways for biodegradation of synthetic chemicals comprise enzymes that are closely related to those for natural compounds. Thus, they are widely distributed and often difficult to distinguish. In contrast, the genes encoding aerobic DNAN degradation offer a unique target for development of molecular tools to evaluate populations. Here, we report a clear relationship between biomarker gene abundances with DNAN-dependent growth of JS1661 under aerobic conditions. The amplicon is of ideal length for a simple qPCR approach applicable for both liquid and soil samples. The *dnhA-B* genes of strain JS1661 are unique, so this qPCR primer design allows for sensitive and specific detection of microbes known to be responsible for using a major IMC waste stream component as a growth substrate under aerobic conditions.

## Supplementary Material

kuae047_Supplemental_File
