# Supplementary material for: Use of qPCR to monitor 2,4-dinitroanisole degrading bacteria in water and soil slurry cultures
Source: J Ind Microbiol Biotechnol. 2024 Nov 23;51:kuae047. doi: 10.1093/jimb/kuae047 (PMC11631463; doi:10.1093/jimb/kuae047)
Supplement: kuae047_Supplemental_File [file kuae047_supplemental_file.docx]

**Supplemental Tables and Figures**

For Waidner et al., ***Use of qPCR to Monitor 2,4-Dinitroanisole Degrading Bacteria in Water and Soil Slurry Cultures***

**Supplemental Table S1.** Amino acid matches to sequences of DNHA and DNHB hydrolases encoded by *dnhA-B* of *Nocardioides* sp. strain JS1661. Closest matches from genomes or metagenomes with >50% and >40% amino acid identity, for DNHA and DNHB, respectively are shown. From SwissProt/UniProt databases (indicated by ***SP** in the Descriptions), only the two closest matches to each DNHA and DNHB are provided. Note, lengths of JS1661 proteins DNHA and DNHB are **328** AA, and **318** AA, respectively. Sequences that are included in the tree, **Supplemental Figure S3**, are indicated by two asterisks (**) adjacent to the accession numbers.

| **Description^a^** | **Accession (length)^b^** | **QC (%)^c^** | **% ident^d^** | **Source** | **Reference^e^** |
| --- | --- | --- | --- | --- | --- |
| **DNHA** | | | | | |
| MBL fold M-H [*Nocardia testacea*] | ******WP_157181917.1 (293) | 88 | 66 | medical patient | NR |
| MBL fold M-H [*Woeseiaceae* sp.] | HEX6259451.1 (206) | 51 | 53 | soil | MAG, TPA (Ma et al. 2023) |
| MBL fold M-H [*Thermoleophilaceae* sp.] | ******HXD59087.1 (293) | 88 | 52 | soil | MAG, TPA (Ma et al. 2023) |
| MBL fold M-H [*Solirubrobacterales* sp.] | ******MBV9423380.1 (304) | 88 | 51 | rhizosphere metagenome | MAG (Camargo et al. 2023) |
| MBL fold M-H [*Solirubrobacterales* sp.] | MBV9003485.1 (304) | 88 | 51 | rhizosphere metagenome | MAG (Camargo et al. 2023) |
| MBL fold M-H [*Gammaproteobacteria* sp.] | ******MDH3511157.1 (311) | 90 | 51 | estuarine surface sediment | MAG |
| hyp. pr. [Candidatus *Thiodiazotropha* sp.] | MCU7857005.1 (200) | 58 | 50 | mollusc gill, *Lucinoma borealis* | MAG |
| MBL fold M-H [*Gammaproteobacteria* sp.] | ******MDJ0929401.1 (309) | 90 | 50 | marine sediment | MAG |
| MBL fold M-H [*Solirubrobacterales* sp.] | ******MBV8431602.1 (304) | 91 | 50 | rhizosphere metagenome | MAG (Camargo et al. 2023) |
| specific secondary-alkylsulfatase (Pisa1) ***SP** | ******F8KAY7.1 (663) | 17 | 36 | *Pseudomonas* sp. | (Knaus et al. 2012) |
| primary-alkylsulfatase (Type III) ***SP** | ******P32717.2 (661) | 17 | 36 | *Escherichia coli* strain K12 | (Liang et al. 2014) |

Table continued, next page.

**Supplemental Table S1, ctd.**

| **Description^a^** | **Accession (length)^b^** | **QC (%)^c^** | **% ident^d^** | **Source** | **Reference^e^** |
| --- | --- | --- | --- | --- | --- |
| **DNHB** | | | | | |
| hyp. pr. [*Arthrobacter* sp. efr-133-R2A-63] | WP_284762100.1 (116) | 33 | 77 | root/leaf, *Arabidopsis thaliana* | NR |
| hyp. pr. [*Nocardia testacea*] | ******WP_039827746.1 (326) | 88 | 42 | medical patient | NR |
| hyp. pr. [*Thermoleophilaceae* sp.] | ******HXD59086.1 (280) | 88 | 42 | soil metagenome | MAG, TPA: (Ma et al. 2023) |
| hyp. pr. [*Solirubrobacterales* sp.] | ******MBV8431601.1 (199) | 61 | 41 | rhizosphere metagenome | MAG (Camargo et al. 2023) |
| primary-alkylsulfatase (Type III) *SP | ******P32717.2 (661) | 55 | 27 | *E. coli* strain K12 | (Liang et al. 2014) |
| specific secondary-alkylsulfatase (Pisa1) *SP | ******F8KAY7.1 (663) | 32 | 29 | *Pseudomonas* sp. | (Knaus et al. 2012) |

^A^ MBL-fold M-H, metallo-beta-lactamase (MBL) fold metallo-hydrolase; hyp. pr., hypothetical protein; *SP, biochemically characterized protein (SwissProt, UniProt).

^B^ Length of protein sequence (amino acids);

^C^ QC, query coverage (percent), percent of DNHA or DNHB length (in AA) aligning with the match.

^D^ % ident., percent of amino acids in the alignment that are identical between DNHA or DNHB with the match.

^E^ Reference column is left blank if the sequence is not associated with a publication. NR, non-redundant protein record; MAG, Metagenome Assembled Genome; TPA, Third-Party Annotation.

**Supplemental Table S2.** Most probable number (MPN) and confidence intervals (CI) to estimate cell density in the liquid culture experiments. Units, most probable number of viable cells (MPN) per mL of liquid culture.

| **Time (hr)** | **MPN (+/- CI),**  **assay measurement #1** | | | **MPN (+/- CI),**  **assay measurement #2** | | |
| --- | --- | --- | --- | --- | --- | --- |
|  | MPN/mL | Upper | Lower | MPN/mL | Upper | Lower |
| **Flask culture #1** | | | | | | |
| **0** | 6.92E+04 | 1.63E+05 | 3.02E+04 | 4.41E+04 | 1.01E+05 | 1.83E+04 |
| **9** | 2.71E+05 | 5.40E+05 | 9.53E+04 | 2.71E+05 | 5.40E+05 | 9.53E+04 |
| **11** | 5.75E+05 | 1.39E+06 | 2.49E+05 | 7.23E+05 | 1.75E+07 | 3.15E+05 |
| **13** | 7.23E+05 | 1.75E+07 | 3.15E+05 | 9.34E+05 | 2.09E+06 | 3.99E+05 |
| **16** | 6.02E+06 | 1.50E+07 | 2.59E+06 | 4.80E+06 | 1.17E+07 | 2.02E+06 |
| **18** | 3.17E+06 | 6.49E+06 | 1.13E+06 | 3.17E+06 | 6.49E+06 | 1.13E+06 |
| **Flask culture #2** | | | | | | |
| **0** | 5.58E+04 | 1.34E+05 | 2.41E+04 | 1.53E+05 | 3.14E+05 | 5.99E+04 |
| **9** | 3.17E+05 | 6.49E+05 | 1.13E+05 | 9.34E+05 | 2.09E+06 | 3.99E+05 |
| **11** | 7.23E+05 | 1.70E+06 | 3.15E+05 | 7.23E+05 | 1.70E+06 | 3.15E+05 |
| **13** | 1.16E+06 | 2.50E+06 | 4.82E+05 | 1.24E+06 | 2.64E+06 | 5.08E+05 |
| **16** | 3.17E+06 | 6.49E+06 | 1.13E+06 | 3.17E+06 | 6.49E+06 | 1.13E+06 |
| **18** | 4.53E+06 | 1.08E+07 | 1.87E+06 | 3.17E+06 | 6.49E+06 | 1.13E+06 |

**Supplemental Table S3.** Most probable number (MPN) and confidence intervals (CI) to estimate cell density in the soil slurry experiments. Units, most probable number of viable cells per mL of soil slurry.

| **Time (hr)** | **MPN (+/- CI),**  **assay measurement #1** | | | **MPN (+/- CI),**  **assay measurement #2** | | |
| --- | --- | --- | --- | --- | --- | --- |
|  | MPN/mL | Upper | Lower | MPN/mL | Upper | Lower |
| **Flask slurry #1** | | | | | | |
| **0** | 4.41E+04 | 1.01E+05 | 1.83E+04 | 1.68E+05 | 3.43E+05 | 6.44E+04 |
| **4** | 3.17E+05 | 6.49E+05 | 1.13E+05 | 9.34E+04 | 2.09E+05 | 3.99E+04 |
| **9** | 3.17E+05 | 6.49E+05 | 1.13E+05 | 2.83E+05 | 5.66E+05 | 9.97E+04 |
| **13** | 5.18E+05 | 1.24E+06 | 2.22E+05 | 6.99E+05 | 1.65E+06 | 3.04E+05 |
| **16** | 2.06E+06 | 4.13E+06 | 7.51E+05 | 3.81E+06 | 8.28E+06 | 1.48E+06 |
| **18** | 9.41E+05 | 2.11E+06 | 4.02E+05 | 4.69E+05 | 1.10E+06 | 1.97E+05 |
| **20** | 7.70E+06 | 1.86E+07 | 3.30E+06 | 3.17E+06 | 6.49E+06 | 1.13E+06 |
| **Flask slurry #2** | | | | | | |
| **0** | 7.23E+04 | 1.70E+05 | 3.15E+04 | 5.75E+04 | 1.39E+05 | 2.49E+04 |
| **4** | 1.86E+05 | 3.73E+05 | 6.95E+04 | 1.24E+05 | 2.64E+05 | 5.08E+04 |
| **9** | 5.75E+05 | 1.39E+06 | 2.49E+05 | 5.58E+04 | 1.34E+05 | 2.41E+04 |
| **13** | 1.44E+06 | 2.99E+06 | 5.70E+05 | 5.75E+05 | 1.39E+06 | 2.49E+05 |
| **16** | 2.29E+06 | 4.57E+06 | 8.17E+05 | 2.76E+06 | 5.53E+06 | 9.64E+05 |
| **18** | 6.02E+06 | 1.50E+07 | 2.59E+06 | 7.70E+06 | 1.86E+07 | 3.30E+06 |
| **20** | 1.40E+07 | 3.03E+07 | 5.48E+06 | 7.70E+06 | 1.86E+07 | 3.30E+06 |

**Supplemental Table S4.** MIQE checklist. The relevant information as recommended by (Bustin et al. 2009) is provided. The checklist items pertaining to RT-qPCR or dPCR are not included.

| **Item to check ^A^** | **Importance** | **Location of the information in this manuscript ^B^** |
| --- | --- | --- |
| **Experimental design** | | |
| Definition of experimental and control groups | E | M&M |
| Number within each group | E | M&M |
| Assay carried out by the investigator’s laboratory? | D | Yes, investigator’s laboratory |
| Acknowledgment of authors’ contributions | D | In cover letter (“declarations” section) sent with initial submission |
| **Sample** | | |
| Description | E | M&M |
| Volume/mass of sample processed | E | M&M |
| Microdissection or macrodissection | E | N/A |
| Processing procedure | E | M&M |
| If frozen, how and how quickly? | E | M&M |
| If fixed, with what and how quickly? | E | N/A |
| Sample storage conditions and duration (especially for FFPEb samples) | E | M&M |
| **Nucleic acid extraction** | | |
| Procedure and/or instrumentation | E | M&M |
| Name of kit and details of any modifications | E | M&M |
| Source of additional reagents used | D | M&M |
| Details of DNase or RNase treatment | E | M&M |
| Contamination assessment (DNA or RNA) | E | Supplemental Figure S5 |
| Nucleic acid quantification | E | M&M |
| Instrument and method | E | M&M |
| Purity (A260/A280) | D | N/A |
| Yield | D | Not analyzed |
| Electrophoresis traces | D | Supplemental Figure S2A |
| Inhibition testing (Cq dilutions, spike, or other) *Note, we use the C_T_ term in place of Cq.* | E | Supplemental Figure **S5**, |
| **qPCR target information** | | |
| Gene symbol | E | Intro, Supplemental Table S1 |
| Sequence accession number | E | M&M, Supplemental Figure S1 |
| Location of amplicon | D | M&M, Supplemental Figure S1B |
| Amplicon length | E | M&M, Supplemental Figure S1B |
| In silico specificity screen (BLAST, and so on) | E | Results, Supplemental Table S1 |
| Pseudogenes, retropseudogenes, or other homologs? | D | Results, Supplemental Table S1 |
| Sequence alignment | D | Raw alignments are not provided, but alignments were done to make the tree figure. Also, in M&M, we describe how the sequenced amplicon was aligned with the accession # record sequence. |
| Secondary structure analysis of amplicon | D | Not analyzed |
| **qPCR oligonucleotides** | | |
| Primer sequences | E | Table 1 |
| RTPrimerDB identification number | D | N/A |
| Probe sequences | D | N/A |
| Location and identity of any modifications | E | No modifications |
| Manufacturer of oligonucleotides | D | M&M |
| Purification method | D | M&M |
| **Item to check ^A^** | **Importance** | **Location of the information in this manuscript ^B^** |
| **qPCR protocol** | | |
| Complete reaction conditions | E | M&M |
| Reaction volume and amount of cDNA/DNA | E | M&M |
| Primer, (probe), Mg2_, and dNTP concentrations | E | M&M |
| Polymerase identity and concentration | E | M&M |
| Buffer/kit identity and manufacturer | E | M&M |
| Exact chemical composition of the buffer | D | Not available |
| Additives (SYBR Green I, DMSO, and so forth) | E | SYBR Green I, M&M |
| Manufacturer of plates/tubes and catalog number | D | Not provided |
| Complete thermocycling parameters | E | M&M |
| Reaction setup (manual/robotic) | D | Manual. |
| Manufacturer of qPCR instrument | E | M&M |
| **qPCR validation** | | |
| Evidence of optimization (from gradients) | D | Not provided |
| Specificity (gel, sequence, melt, or digest) | E | Results, Supplemental Table S1, Supplemental Figure **S2A** and Supplemental Figure S3C, S3D, S3E |
| For SYBR Green I, Cq of the NTC | E | M&M and Results |
| Calibration curves with slope and y intercept | E | Supplemental Figure S2B |
| PCR efficiency calculated from slope | E | Supplemental Figure S2B |
| CIs for PCR efficiency or SE | D | Not determined |
| r2 of calibration curve | E | Supplemental Figure S2B |
| Linear dynamic range | E | Results and Supplemental Figure S2 |
| Cq variation at LOD (sensitivity, lower limit of detection) | E | Results and Supplemental Figure S2 |
| CIs throughout range | D | Not determined |
| Evidence for LOD | E | Results |
| If multiplex, efficiency and LOD of each assay | E | N/A |
| **Data analysis** | | |
| qPCR analysis program (source, version) | E | M&M |
| Method of Cq determination | E | M&M |
| Outlier identification and disposition | E | M&M and Results |
| Results for NTCs | E | M&M and Results |
| Number and concordance of biological replicates | D | M&M and Results |
| Repeatability (intraassay variation) | E | Results |
| Reproducibility (interassay variation, CV) | D | Not provided |
| Power analysis | D | Not provided |
| Statistical methods for results significance | E | N/A |
| Software (source, version) | E | As above, qPCR analysis program |
| Cq or raw data submission with RDML | D | Not provided. |

**^A^** Line items from the original MIQE table in Bustin et al. (2009) that are specific to RT-qPCR, and hence not relevant to this study, were excluded from the table provided here. The entire section entitled “Reverse Transcription” was also excluded. Additional specific line items excluded are:

- RNA integrity: method/instrument
- RIN/RQI or Cq of 3_ and 5_ transcripts
- Location of each primer by exon or intron (if applicable)
- What splice variants are targeted?
- Justification of number and choice of reference genes
- Description of normalization method
- Number and stage (reverse transcription or qPCR) of technical replicates

**^B^** Location of information in the manuscript: Intro = Introduction; Results = Results/Discussion section; M&M = Materials and Methods section; or specific figures and/or tables are listed when the information is, in part, provided in a figure or table. N/A, not applicable.

**
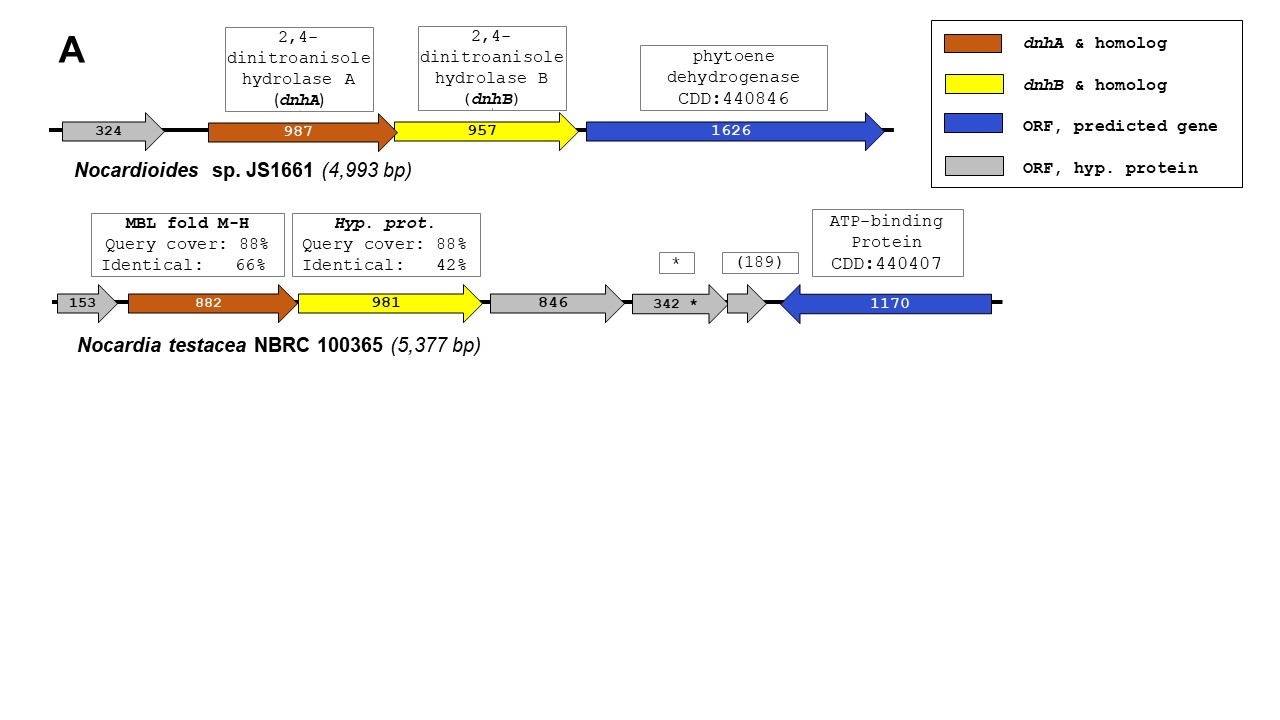


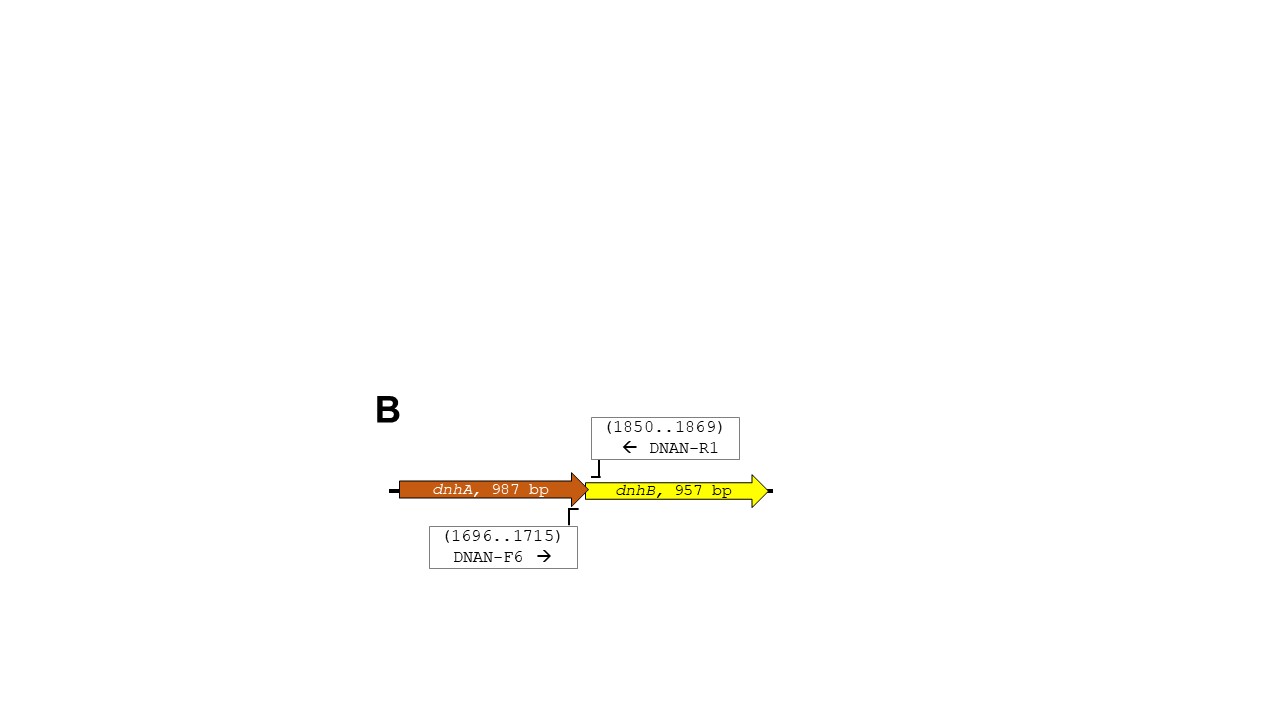
**

**Supplemental Figure S1.** Segment of JS1661 genomic DNA and location of qPCR primers. Panel A, genomic context of the DNHA and DNHB homologs in the *Nocardia testacea* strain NBRC 100365 whole genome shotgun sequence (accession # GCA_000308775.1), from genome assembly ASM30877v1. The open reading frame (ORF) arrows are colored as follows: grey, hypothetical protein; brown and yellow, *dnhA* and *dnhB* or homologs; and blue, annotated based on sequence homology as a predicted protein (name and CDD of protein provided in box above the arrow). Numbers inside or just above the ORF arrows indicate gene length in nucleotides. *The 96,430 bp contig containing the portion shown is available for download at:* [*https://www.ncbi.nlm.nih.gov/datasets/genome/GCF_000308775.1/*](https://www.ncbi.nlm.nih.gov/datasets/genome/GCF_000308775.1/)*.* Asterisk symbol above the grey open reading frame (ORF) arrows corresponding to hypothetical proteins in *Nocardia testacea* indicate frame-shift. Panel B, location of qPCR primers matching to the junction of JS1661 *dnhA* and *dnhB* genes. Position numbering is shown as in the genomic contig in Panel A (and also see accession #[KM213001.1](https://www.ncbi.nlm.nih.gov/nuccore/KM213001.1?report=GenBank)). Positions #1696- 1869 are amplified with the primer pair in **Table 1**.Two additional forward primers and two additional reverse primers were initially tested, but only the pair with the highest sensitivity while maintaining specificity (see **Supp. Figure S2**) is shown. Abbreviations: MBL fold M-H, MBL-fold M-H, metallo-beta-lactamase (MBL) fold metallo-hydrolase; hyp. pr., hypothetical protein.


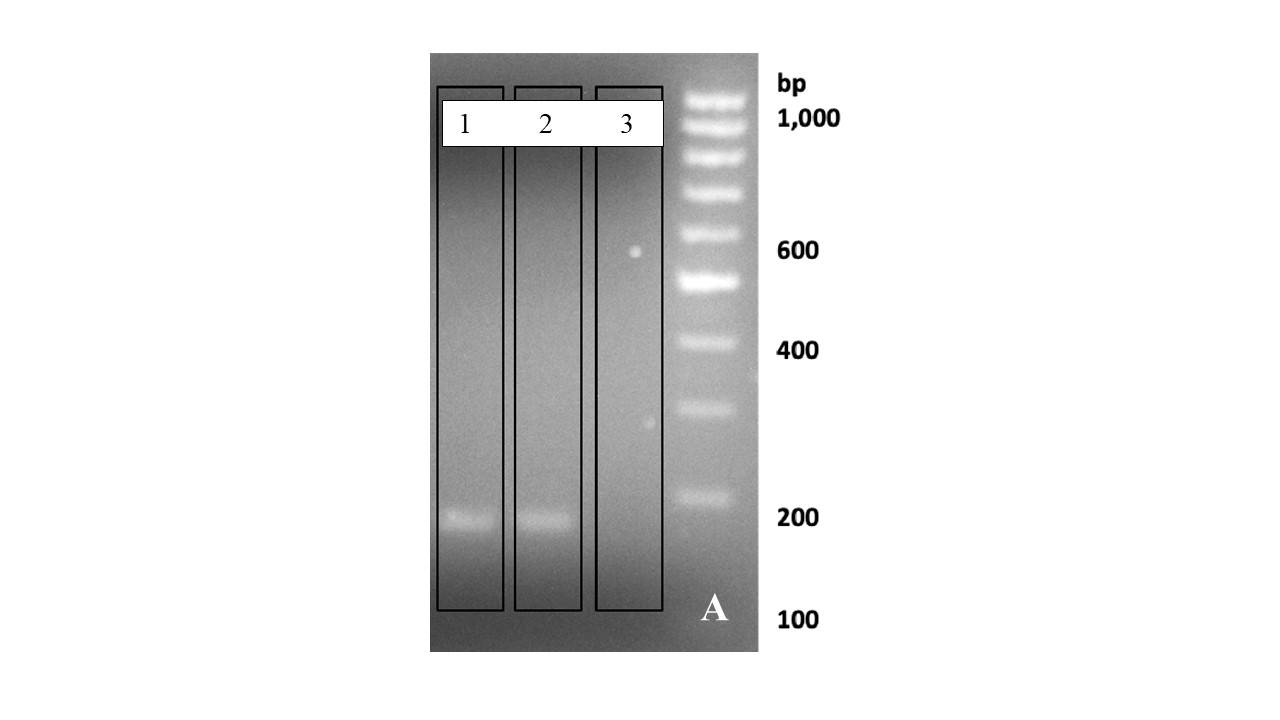
 **
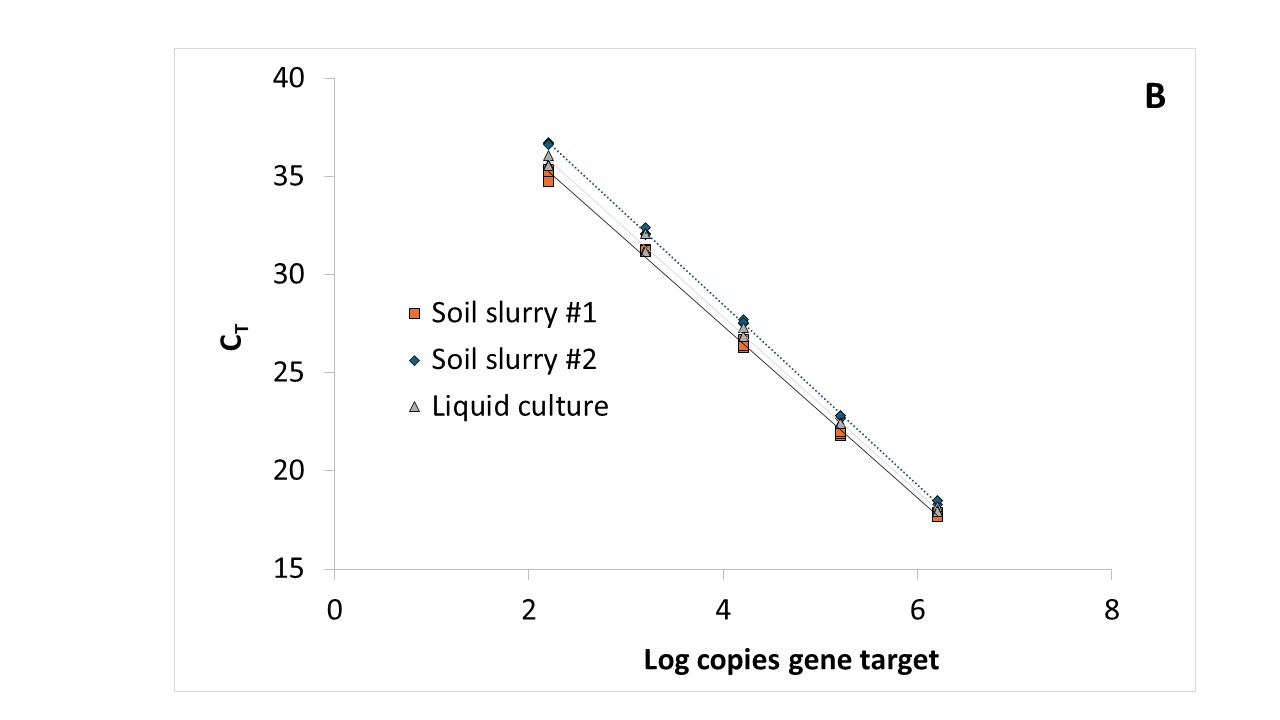
**

**Supplemental Figure S2.** Specificity of qPCR primer pair DNAN-F6 and DNAN-R1and range of standard curves. Panel A, amplicon (174 bp) is a product of PCR from the areas flanking the end of *dnhA* and the start of *dnhB.* Gel lanes show amplicons resulting from PCR using different amounts of purified JS1661 genomic DNA used as the template: lane 1, 2.5 ng DNA; lane 2, 5.0 ng DNA; lane 3, 0.0 ng (water, no-template control). DNA size ladder indicators (in bp) are provided. Panel B, qPCR standard curves with genomic DNA from strain JS1661. Amount of DNA in the standard DNA wells ranged from 0.05 pg to 10.0 ng per well, corresponding to 160 to 1.60 x 10^6^ copies per reaction. The calculated LoD of the assay from replicate standard curves, including 24 wells containing the lowest concentration of the DNA standard, was 38.19 copies per reaction, with a confidence interval of 95%. For each of the DNA concentrations, values for three individual well replicates are plotted. Fits to the linear lines (R^2^ values) and calculated qPCR efficiency for each of the slopes from standard curves in each of the qPCR plates ranged from 0.9984 to 0.9996, and 65.9% to 68.9%, respectively. C_T_: cycle threshold value. Panels C, D, and E: Melt curves of experimental sample DNA and DNA in standard curves for each of three plates in panels (C) liquid culture DNA samples, (D) one plate containing soil slurry culture DNA samples, and (E) another plate containing soil slurry culture DNA samples. In each, the melt curves from the no-template control qPCR (NTC) are also shown separately.

Supplemental Figure S2 panels C, D, and E are continued, starting on the following page.

**Supplemental Figure S2, continued.**

**S2C**


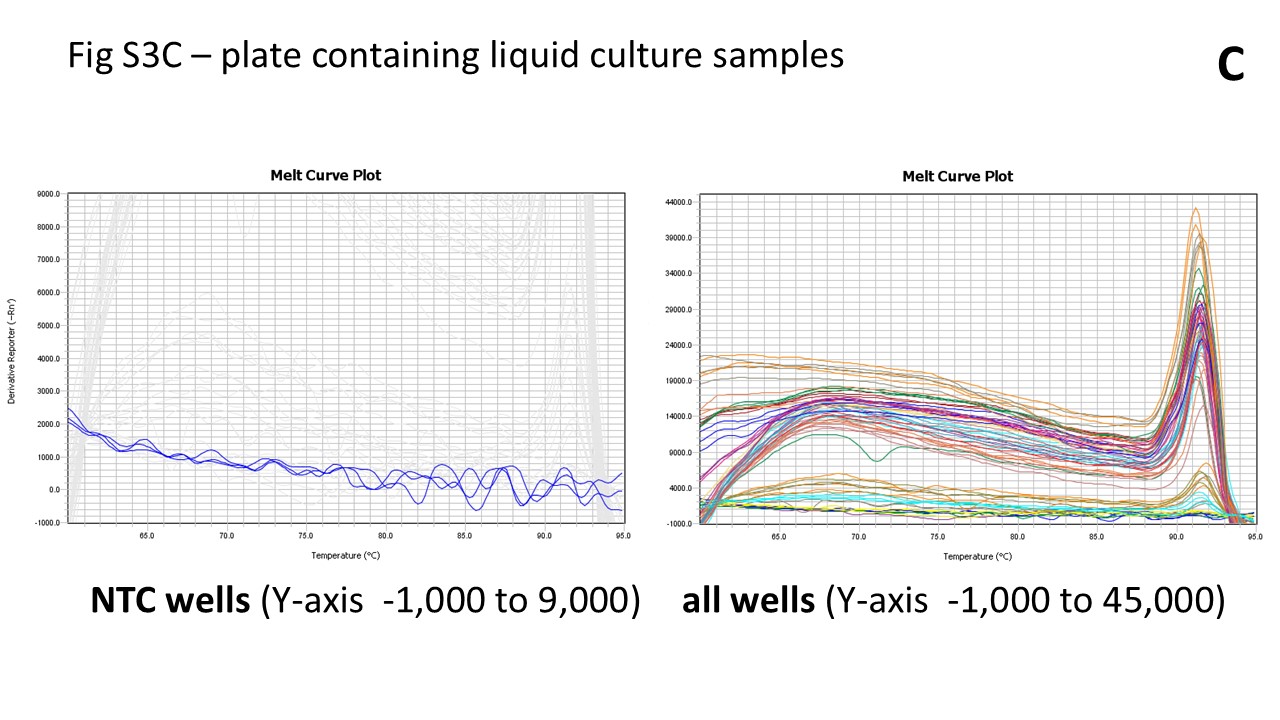


**Supplemental Figure S2, continued.**

**S2D**


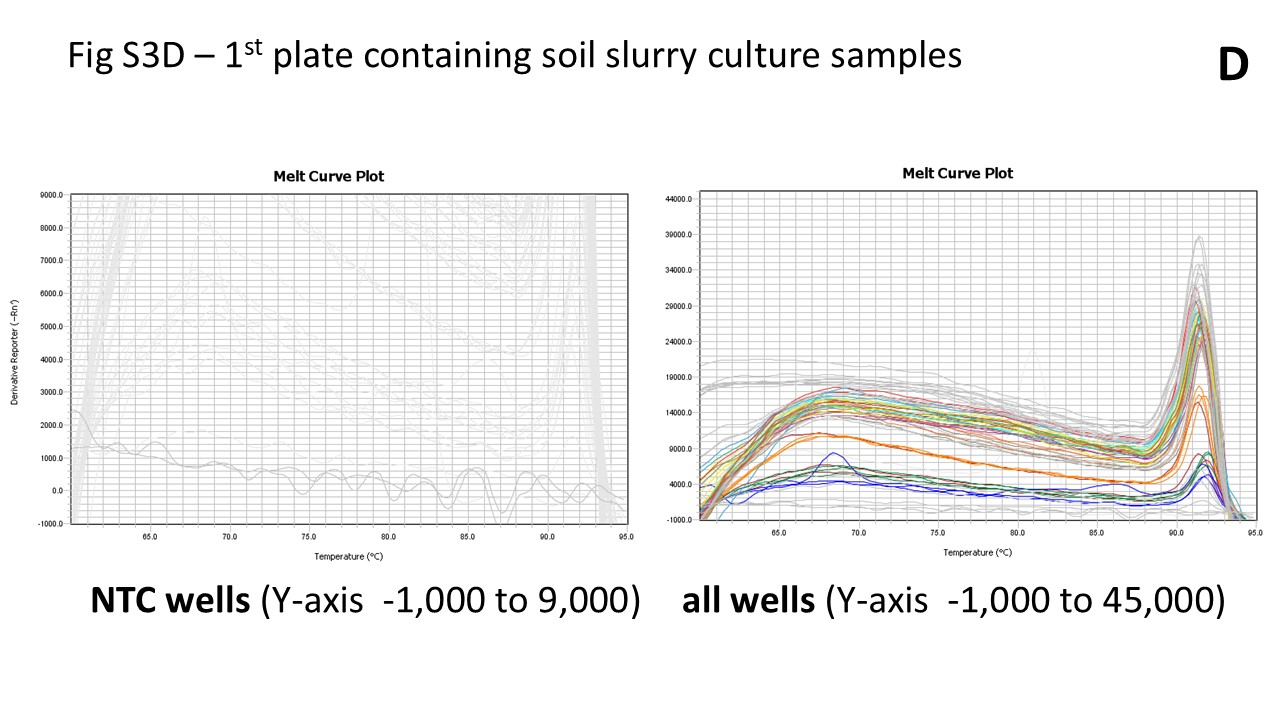


**Supplemental Figure S2, continued.**

**S2E**


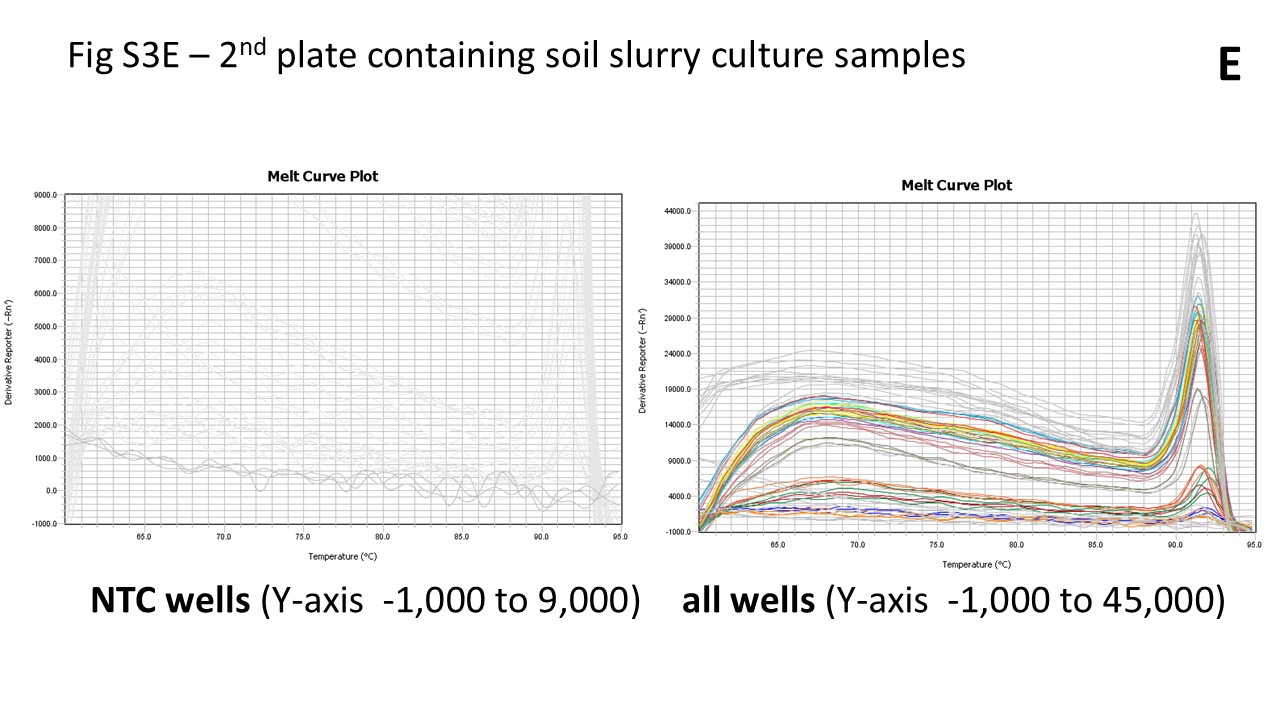


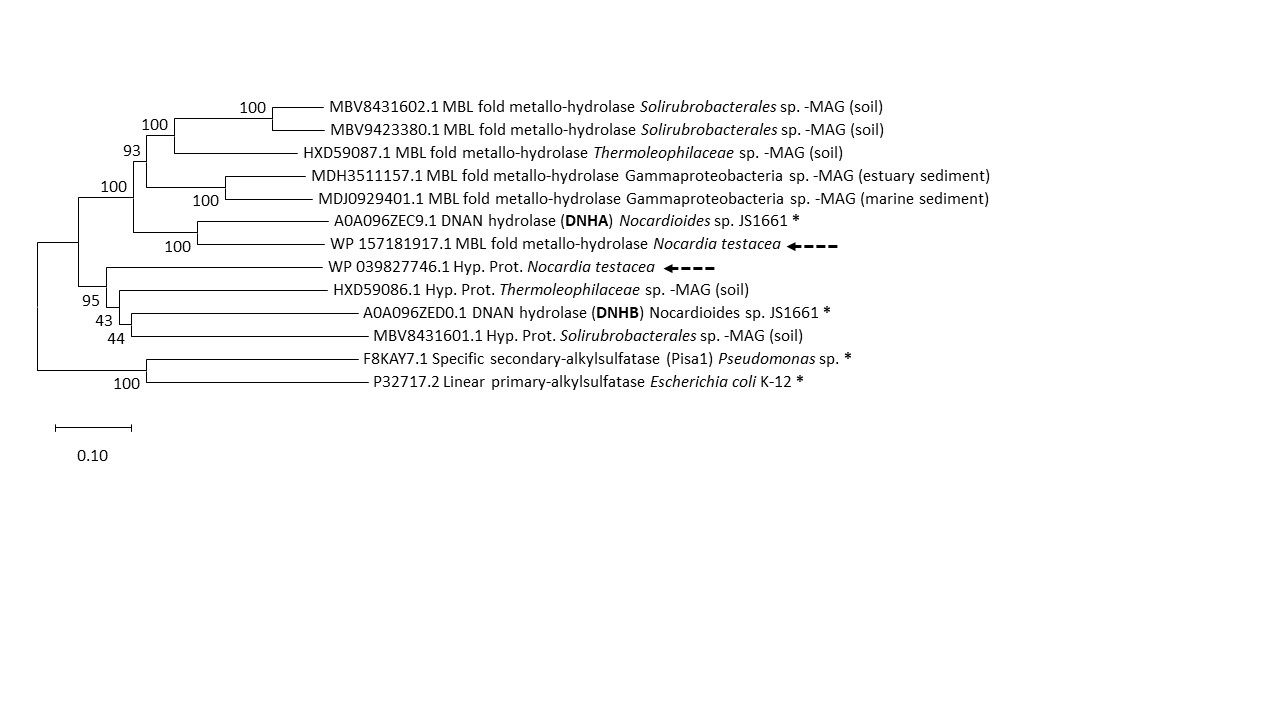


**Supplemental Figure S3.** Neighbor-joining tree of full-length amino acid sequences of DNHA, DNHB and homologs. In this alignment and distance calculation, there were a total of 674 positions in the final dataset. The alignment [MEGA-X, (Kumar et al. 2018)] was used to calculate p-distance (Nei et al. 2000) with pairwise deletion of gaps. In the Neighbor-Joining tree (Saitou and Nei 1987), numbers adjacent to nodes represent the number of trees resulting from bootstrap analysis with 100 replicates (Felsenstein 1985). Branch lengths are drawn to scale, with distance calculated in units of the number of amino acid differences per site. The accession number for each sequence is provided, followed by the description. A single asterisk (**“ * ”**) indicates AA sequences of biochemically-characterized protein sequences from the SwissProt/UniProt database. Dashed arrows point to homologous sequences from the genome of the cultured bacterium *Nocardia testacea,* strain NBRC 100365 (see **Supplemental Figure S1**). MAG, Metagenome Assembled Genome; Hyp. Prot, hypothetical protein sequence.

**
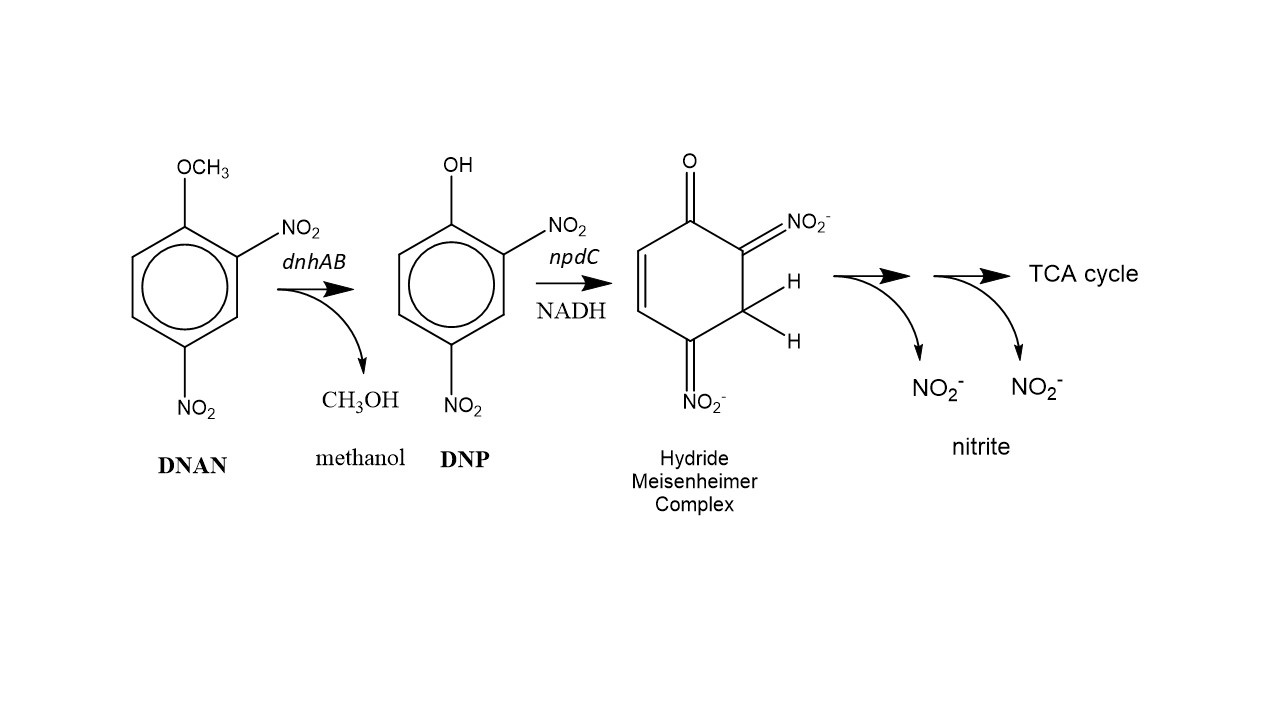
**

**Supplemental Figure S4.** Proposed pathway of DNAN mineralization by *Nocardioides* sp. strain JS1661 via a Hydride Meisenheimer complex. TCA cycle refers to the tricarboxylic acid cycle (Fida et al. 2014)(Fida et al 2014). Genes designated *dnhAB* encode the two component DNAN demethylase and *npdC* encodes the putative hydride transferase.

**Supplemental Figure S5.** PCR inhibitor assessment with DNA samples isolated from soil slurry culture time points. The calculated copies per mL are provided for DNA samples isolated from soil slurry culture#2, at time points 0, 4, 9, 13, 16, 18, and 20 hr of growth. C_T_ values were used to calculate gene copies in DNA assessed in qPCR, including both undiluted DNA and DNA diluted 1/10 with T_10_E_1_ (10 mM Tris, 1 mM EDTA). Throughout all time points assessed, the average ratio of calculated copies from undiluted DNA to copies calculated from qPCR on DNA diluted 1/10 was ~8-fold.

**Supplemental Information references cited**

Bustin SA, Benes V, Garson JA, et al (2009) The MIQE guidelines: minimum information for publication of quantitative real-time PCR experiments. Clin Chem 55:611–622. https://doi.org/10.1373/clinchem.2008.112797

Camargo AP, de Souza RSC, Jose J, et al (2023) Plant microbiomes harbor potential to promote nutrient turnover in impoverished substrates of a Brazilian biodiversity hotspot. ISME J 17:354–370. https://doi.org/10.1038/s41396-022-01345-1

Felsenstein J (1985) Confidence limits on phylogenies: An approach using the bootstrap. Evol Int J Org Evol 39:783–791. https://doi.org/10.1111/j.1558-5646.1985.tb00420.x

Fida TT, Palamuru S, Pandey G, Spain JC (2014) Aerobic biodegradation of 2,4-dinitroanisole by Nocardioides sp. strain JS1661. Appl Environ Microbiol 80:7725–7731. https://doi.org/10.1128/AEM.02752-14

Knaus T, Schober M, Kepplinger B, et al (2012) Structure and mechanism of an inverting alkylsulfatase from Pseudomonas sp. DSM6611 specific for secondary alkyl sulfates. FEBS J 279:4374–4384. https://doi.org/10.1111/febs.12027

Kumar S, Stecher G, Li M, et al (2018) MEGA X: Molecular evolutionary genetics analysis across computing platforms. Mol Biol Evol 35:1547–1549. https://doi.org/10.1093/molbev/msy096

Liang Y, Gao Z, Dong Y, Liu Q (2014) Structural and functional analysis show that the Escherichia coli uncharacterized protein YjcS is likely an alkylsulfatase. Protein Sci Publ Protein Soc 23:1442–1450. https://doi.org/10.1002/pro.2528

Ma B, Lu C, Wang Y, et al (2023) A genomic catalogue of soil microbiomes boosts mining of biodiversity and genetic resources. Nat Commun 14:7318. https://doi.org/10.1038/s41467-023-43000-z

Nei M, Kumar S, Nei M, Kumar S (2000) Molecular Evolution and Phylogenetics. Oxford University Press, Oxford, New York

Saitou N, Nei M (1987) The neighbor-joining method: a new method for reconstructing phylogenetic trees. Mol Biol Evol 4:406–425. https://doi.org/10.1093/oxfordjournals.molbev.a040454
